# Development of a Brief Multicultural Version of the Test of Mobile Phone Dependence (TMDbrief) Questionnaire

**DOI:** 10.3389/fpsyg.2016.00650

**Published:** 2016-05-25

**Authors:** Mariano Chóliz, Lourdes Pinto, Sukanya S. Phansalkar, Emily Corr, Ayman Mujjahid, Conni Flores, Pablo E. Barrientos

**Affiliations:** ^1^Faculty of Psychology, University of ValenciaValencia, Spain; ^2^Universidad Autónoma de YucatánMérida, Mexico; ^3^Savitribai Phule Pune UniversityPune, India; ^4^Dún Laoghaire Institute of Art, Design and TechnologyDublin, Ireland; ^5^University of Management and TechnologyLahore, Pakistan; ^6^Universidad Nacional de San AgustinArequipa, Peru; ^7^Universidad del Valle de GuatemalaGuatemala, Guatemala

**Keywords:** mobile dependence, technological addictions, mobile phone use, cross-cultural studies, gender differences

## Abstract

The Test of Mobile Phone Dependence (TMD) questionnaire (Chóliz, 2012) evaluates the main features of mobile phone dependence: tolerance, abstinence syndrome, impaired impulse control, associated problems, excessive use, etc.

**Objective:** The objective of this study was to develop a multicultural version of the TMD (TMDbrief) adapted to suit the novel communication tools of smartphones.

**Procedure:** In this study, the TMD was completed by 2,028 young respondents in six distinct world regions: Southern Europe, Northwest Europe, South-America, Mesoamerica, Pakistan, and India.

**Results:** Psychometric analysis of the reliability of the instrument and factor analysis were performed to adapt the TMDbrief for use in these regions. Differences among regions with respect to TMD Mobile Phone Dependence scores were obtained.

**Conclusion:** A brief questionnaire for the evaluation of mobile phone addiction in cross-cultural studies was successfully developed.

## Introduction

The use of mobile phones has been increasing exponentially in the majority of the world’s countries, reaching a state in which there are almost as many mobile phones as there are people (Ict Data and Statistics Division, 2015). This is the case in India and Pakistan, which have attained penetration index values (phones per inhabitant rates) of approximately 80% (Pakistan Telecommunication Authority [PTA], 2015; Telecom Regulatory Authority of India [TRAI], 2015). Moreover, in many countries, there are already more mobile phones than there are people, as is the case in Ireland (Thinkhouse, 2015), Spain (Observatorio Nacional de las Telecomunicaciones y de la Sociedad de la Información [ONTSI], 2015), Guatemala (iLifeBelt, 2015), and Peru (Ministerio de Transportes y Comunicaciones de Perú [MTC], 2015).

From the outset, the mobile phone was designed not only as a means by which to talk to other people in any location but also as a multifunctional electronic device with many applications previously supported only by other, specialized devices, e.g., use as a photo camera, video recorder or player, alarm clock, games console, and so on. Furthermore, the mobile phone has many attributes that are highly attractive to young people and teenagers that serve to encourage its use. These include (Chóliz, 2010a): (a) the reinforcement of personal autonomy, especially with regard to parents (Oksman and Turtiainen, 2004); (b) the provision of identity and prestige in the context of peer relationships (Oksman and Rautiainen, 2003; Ling and Pedersen, 2006), a facet that is quite evident with the newest and most fashionable models (Katz and Sugiyama, 2005); (c) the introduction of major technological innovations, allowing adolescents to demonstrate a particular inclination or skill (Subrahmanyam et al., 2001); (d) a source of fun and entertainment; and (e) support for the establishment and maintenance of interpersonal relationships (Taylor and Harper, 2003). This function is of paramount importance for young children and adolescents. In the course of a few years, the manner in which mobile phones are used has changed. Specifically, the development of numerous computer applications, or “apps,” has transformed the mobile phone into a much more versatile and faster device with which one can perform many daily activities more efficiently. However, the greatest revolution in mobile phones occurred with the appearance of the Internet on mobile phone platforms, which has drastically changed patterns of use. Now, mobiles serve as computer terminals through which it is possible to use any of the functions of the Internet, from any location. The mobile phone has essentially become an intelligent, multifunctional device, i.e., a “smart” phone, a new class of mobile phones that provide integrated communication, computing, and mobile sector services (Sarwar and Soomro, 2013).

The transformation of the “portable phone” into a “smart mobile device” (many times without using the word “phone”) has been a revolution not only in terms of expanding the number of functions of the mobile phone but also specifically in terms of its most basic function, which remains interpersonal communication. Currently, mobiles offer the possibility of broad, fast, and multifunctional communication with others due to the appearance of instant messaging (IM) software such as WhatsApp, Line, Snatchap, and Viber, whereas before (i.e., only a few years ago) communication was restricted to phone conversations or Short Message Service (SMS) texts.

Instant messaging systems allow communication to be more accessible and attractive. Currently, the use of such systems is heavily promoted, and patterns of interpersonal communication are changing toward a model framed by the readiness of the recipient and immediacy of response. In youths and adolescents, for whom interpersonal communication is so important, this can lead to excessive use, misuse, and in the most severe cases, dependence on smartphones.

Hence, despite the many interesting and useful functions that the mobile phone fulfills in modern society, maladaptive use of mobile phones has been identified, and has been linked with psychological dysfunction (Hamblin et al., 2004; Leena et al., 2005; Billieux et al., 2008; Yen et al., 2009; Yang et al., 2010), health problems (Frey, 1998; Croft et al., 2002; Hardell et al., 2007; Lajunen et al., 2007; Sahin et al., 2013), and even psychiatric disorders (Takao et al., 2009; Demirci et al., 2015). One of the most common problems associated with dysfunctional mobile phone use is the dependence that mobiles can foster in many individuals, particularly in young people, with resulting addiction-related difficulties (Chóliz, 2010b; Koo and Park, 2010; Ahmed et al., 2011).

Among the most characteristic symptoms of mobile dependence are the following (Chóliz, 2010b): (a) excessive use, both in terms of high economic cost and the number of calls and messages; (b) interpersonal problems associated with excessive use; (c) interference with academic or occupational activities; (d) tolerance, i.e., a gradual increase in the amount of use needed to obtain the same level of satisfaction, as well as the need to substitute operative devices with new models that appear on the market; (e) abstinence symptoms, i.e., an urgent need to use a mobile phone after some time has elapsed since its last use, as well as emotional alterations when its use is impeded or made difficult; (f) lack of control, i.e., inability to stop the addictive behavior.

In summary, the use of smartphones is increasing, and the pattern of communication is broadly changing toward one in which mobile phone dependence or addiction is a consequence for some people. This is not a new problem; previous studies have already documented mobile phone addiction, considered to be one of the most important technological addictions, even before the implementation of IM systems based on the Internet (e.g., WhatsApp, Viber, Line, etc.). The novel benefits that IM systems offer actually worsen the problem of addiction because they promote the very factors involved in the dependence process, namely availability, accessibility, immediacy of effects, attractiveness, and so forth.

As noted above, the use of mobile phones is not a local or regional phenomenon. In recent years, there has been increasing interest in the psychological problems associated with the use of mobiles in countries such as China (Leung, 2008; Yen et al., 2009; Yang et al., 2010; Hong et al., 2012), Finland (Oksman and Rautiainen, 2003), Germany (Thomas et al., 2010), Hungary (Mezel et al., 2007), Iran (Alavi et al., 2014), Italy (Martinotti et al., 2011; Pastore and Chóliz, 2011), Japan (Toda et al., 2004; Igarashi et al., 2008), Pakistan (Sahin et al., 2013), South Korea (Ha et al., 2008; Koo, 2009, 2010), Spain (Jenaro et al., 2007; Chóliz et al., 2009; Carbonell et al., 2012), Sweden (Söderqvist et al., 2007), Tunisia (Halayem et al., 2010), the United Arab Emirates (Hashem, 2009), and others. Despite the evident cultural and social differences among young people in different countries, they all use mobile phones in a similar manner, even with respect to specific IM systems such as WhatsApp or Snapchat.

Instruments have been developed in various countries for the purpose of evaluating addiction to mobile phones and the problems associated with their dysfunctional use (Samkange-Zeeb et al., 2004; Bianchi and Phillips, 2005; Toda et al., 2006; Rutland et al., 2007; Chóliz, 2012; Kwon et al., 2013a,b). The mobile phone is currently an integral part of interpersonal relationships and despite the cultural diversity evident across different societies, the use of mobiles among adolescents and youth is a global phenomenon. Nevertheless, few studies have compared mobile phone addiction among young people in culturally diverse regions. Hence, research is needed to explore pertinent differences related to mobile addiction among the youth of different countries.

The objective of this study was to develop a version of the Test of Mobile Phone Dependence (TMD; Chóliz and Villanueva, 2011; Chóliz, 2012), which is a questionnaire that evaluates the main dimensions of mobile phone dependence in adolescence, in order that it can be easily used by part of many clinicians and researchers from different countries. The resulting short questionnaire (“TMDbrief”) could be of use for carrying out cross-cultural studies on mobile phone addiction.

## Materials and Methods

### Participants

The sample consisted of 2,028 young adults between 18 and 27 years of age (1,160 women and 868 men). All participants were graduate or postgraduate students from public or private universities in seven countries representing three different continents (America, Asia, and Europe).

### Measure

The TMD questionnaire (Chóliz and Villanueva, 2011; Chóliz, 2012) was chosen by several researchers interested in this topic due to its good psychometric properties and its appropriateness for investigating problems related to mobile phone addiction in their respective countries. These researchers remained in contact with the author, and data were collected in college settings or by email (via a Google Docs document) in case that they couldn’t respond in the classroom (5.2 percent of all of them).

The questionnaire exhibits good reliability (Cronbach’s α = 0.94), and consists of 22 items grouped into three factors: (a) abstinence, (b) lack of control and problems derived from use, and (c) tolerance and interference with other activities. The TMD was initially developed according to the Diagnostic and Statistical Manual of Mental Disorders (DSM-IV-TR) criteria for dependence disorder. The instrument was previously validated for use in adolescents and consists of 22 items rated on Likert-type scales. The first 10 items are answered on scales ranging from 0 (*never*) to 4 (*frequently*). The 12 remaining items use a scale ranging from 0 (*completely disagree*) to 4 (*completely agree*).

### Procedure

They responded freely and voluntarily to the items on the questionnaire and they had the option of withdrawing their participation at any time. The questionnaire was anonymous, and no data were obtained that would allow any individual to be personally identified. This study was part of a global research study whose ethical protocols and research design were approved by the University of Valencia (Spain) on May 29, 2012.

In each continent, the sample was drawn from two distinct cultural regions. The countries and regions included were as follows: 222 individuals from Ireland (Northwest Europe, English speaking) (115 women, 107 men); 438 from Spain (South Europe, Spanish speaking) (298 women, 140 men); 399 from Peru (South America, Spanish speaking) (200 women, 199 men); 311 from Mexico and Guatemala (Mesoamerica, Spanish speaking) (201 women, 110 men); 399 from Pakistan (Islamic Asia, English speaking) (192 women, 207 men), and 259 from India (Hindu Asia, English and Marathi speaking) (154 women, 105 men). The translation to Marathi has been carried out by part of the Indian co-author of the article, who is Marathi speaker. The Mexican and Guatemalan participants were considered to be from the Mesoamerican region. The Mexican participants were drawn from Yucatán, near Guatemala.

### Analysis

All of the data were analyzed using the SPSS for Windows software package (version 19.0; SPSS, Inc., Chicago, IL, USA). Reliability analyses and an exploratory factor analysis were performed. Principal-components analysis was used to extract the relevant factors, with promax rotation, Kaiser normalization, and a kappa value of 4 applied. Basic and central-tendency descriptive statistics (Pearson bivariate correlations, analyses of variance (ANOVAs) were also performed. Subsequently, several analyses of mean differences among different countries or according to gender were carried out.

## Results

### Reliability and Factor Analysis

For the reliability analysis, Cronbach’s alpha was computed. Items with a corrected homogeneity index >0.5 were selected; when correlations between each of the items and the rest of the scale are ≥0.5, all of the items can be said to measure the same construct (mobile phone dependence, in the present instance). In order to develop a brief scale, we select the more representative items in every factor: (a) scores above 0.70 in the structural matrix, and (b) a global alpha coefficient of the scale higher than 0.80. This results in the current factorial structure. The items selected in this manner were items 4, 6, 9, 13, 14, 15, 16, 17, 18, 20, 21, and 22. The Cronbach’s alpha value for these 12 items was 0.88.

Factor analysis was used to analyze the structure of the questionnaire. Principal-components analysis was used to extract the factors, and Promax normalization with a Kappa value of 4 was used for the rotation. This technique was applied because we assumed *a priori* that the factors were correlated with one another, as the dimensions that constitute the construct of mobile dependence are not independent.

The Bartlett sphericity contrast [χ^2^(66) = 10,677.123; *p* < 0.001] allowed us to reject the null hypothesis that the variables used in the analysis were not correlated in the population from which we extracted the sample, which further allowed us to consider the correlation matrix suitable for factor analysis. Furthermore, the Kaiser–Meyer–Olkin measure of sample adequacy (KMO = 0.92) also indicated that the correlation matrix was adequate for the analysis.

Four factors with eigenvalues greater than 1 were extracted from the factor analysis, which together explained 66.68% of the variance. The first factor explained 44.08% of the variance and was composed of items 13, 15, and 20 from the original questionnaire (Cronbach’s α = 0.81). Based on the content of these items, the first factor was termed *Abstinence*, as it refers both to the discomfort felt when unable to use mobile phones and to the use of these phones to alleviate psychological problems. The second factor, named *Abuse and interference with other activities*, explained a further 9.61% of the variance and comprised three TMD items (4, 6, and 9) (Cronbach’s α = 0.70) that pertain to excessive mobile phone use and the degree of interference that this causes with respect to daily activities. The third factor, *Tolerance*, explained 6.76% of the variance and comprised items 14, 16, and 17 from the original instrument (Cronbach’s α = 0.75); factor III pertains to an escalating need for mobile phone use. Finally, factor IV, *Lack of control*, explained 6.22% of the variance and comprised items 18, 21, and 22 of the original instrument (Cronbach’s α = 0.64); this factor pertains to difficulty stopping mobile phone use.

**Table 1** lists the factors constituting the structural matrix.

**Table 1:** Structure matrix.

Item	Text	Factor I	Factor II	Factor III	Factor IV
13	If my mobile phone were broken for an extended period of time and took a long time to fix, I would feel very bad.	0.885	0.380	0.433	0.391
15	If I don’t have my mobile phone, I feel bad.	0.831	0.362	0.618	0.399
20	I don’t think I could stand spending a week without a mobile phone.	0.789	0.376	0.447	0.512
04	I spend more time than I would like to talking on the mobile phone, sending SMSs, or using WhatsApp.	0.310	0.784	0.415	0.298
06	I have gone to bed later or slept less because I was using my mobile phone.	0.365	0.808	0.311	0.371
09	I use my mobile phone (calls, SMSs, WhatsApp...) in situations where, even though not dangerous, it is not appropriate to do so (eating, while other people are talking to me, etc.).	0.347	0.739	0.433	0.373
14	I need to use my mobile phone more and more often.	0.635	0.456	0.712	0.298
16	When I have my mobile phone with me, I can’t stop using it.	0.433	0.476	0.774	0.433
17	Since I got my mobile phone, I have increased the number of SMSs I send.	0.559	0.374	0.808	0.363
18	As soon as I get up in the morning, the first thing I do is see who has called me on my mobile phone or if someone has sent me an SMS.	0.481	0.533	0.232	0.735
21	When I feel lonely, I use the mobile phone (calls, SMSs, WhatsApp...).	0.453	0.311	0.445	0.814
22	I would grab my mobile phone and send a message or make a call right now.	0.291	0.348	0.653	0.701

**Table 2** provides the correlations among the four factors extracted from the questionnaire. The data show that these four factors are indeed interrelated in a direct and statistically significant manner.

**Table 2:** Factor correlation matrix.

	Abstinence	Abuse, interference with other activities	Tolerance	Lack of control	TMDbrief
Abstinence	–	0.457^∗∗^	0.657^∗∗^	0.577^∗∗^	0.841^∗∗^
Abuse, interference with other activities		–	0.534^∗∗^	0.509^∗∗^	0.751^∗∗^
Tolerance			–	0.582^∗∗^	0.851^∗∗^
Lack of control				–	0.816^∗∗^
*Cronbach’s α = 0.88*					

*^∗∗^significant correlation (*p* < 0.01).*

In addition, the same factor analysis was performed for each country to confirm the factorial structure of the TMDbrief. The same factorial structure was obtained for all countries except India, with the only difference in the results for these countries being the eigenvalues associated with each factor (**Tables 3–8**).

**Table 3:** Factor correlation matrix (Spain).

	Abstinence	Abuse, interference with other activities	Tolerance	Lack of control	TMDbrief
Abstinence	–	0.461^∗∗^	0.561^∗∗^	0.487^∗∗^	0.800^∗∗^
Abuse, interference with other activities		–	0.571^∗∗^	0.511^∗∗^	0.779^∗∗^
Tolerance			–	0.558^∗∗^	0.835^∗∗^
Lack of control				–	0.793^∗∗^
*Cronbach’s α = 0.88*					

*^∗∗^significant correlation (*p* < 0.01).*

**Table 4:** Factor correlation matrix (Mexico/Guatemala).

	Abstinence	Abuse, interference with other activities	Tolerance	Lack of control	TMDbrief
Abstinence	–	0.367^∗∗^	0.593^∗∗^	0.414^∗∗^	0.784^∗∗^
Abuse, interference with other activities		–	0.517^∗∗^	0.435^∗∗^	0.735^∗∗^
Tolerance			–	0.431^∗∗^	0.817^∗∗^
Lack of control				–	0.747^∗∗^
*Cronbach’s α = 0.86*					

*^∗∗^significant correlation (*p* < 0.01).*

**Table 5:** Factor correlation matrix (Peru).

	Abstinence	Abuse, interference with other activities	Tolerance	Lack of control	TMDbrief
Abstinence	–	0.509^∗∗^	0.689^∗∗^	0.613^∗∗^	0.842^∗∗^
Abuse, interference with other activities		–	0.592^∗∗^	0.608^∗∗^	0.800^∗∗^
Tolerance			–	0.609^∗∗^	0.860^∗∗^
Lack of control				–	0.849^∗∗^
*Cronbach’s α = 0.90*					

*^∗∗^significant correlation (*p* < 0.01).*

**Table 6:** Factor correlation matrix (Ireland).

	Abstinence	Abuse, interference with other activities	Tolerance	Lack of control	TMDbrief
Abstinence	–	0.529^∗∗^	0.552^∗∗^	0.503^∗∗^	0.828^∗∗^
Abuse, interference with other activities		–	0.550^∗∗^	0.493^∗∗^	0.798^∗∗^
Tolerance			–	0.422^∗∗^	0.789^∗∗^
Lack of control				–	0.761^∗∗^
*Cronbach’s α = 0.87*					

*^∗∗^significant correlation (*p* < 0.01).*

**Table 7:** Factor correlation matrix (Pakistan).

	Abstinence	Abuse, interference with other activities	Tolerance	Lack of control	TMDbrief
Abstinence	–	0.399^∗∗^	0.585^∗∗^	0.623^∗∗^	0.837^∗∗^
Abuse, interference with other activities		–	0.441^∗∗^	0.341^∗∗^	0.683^∗∗^
Tolerance			–	0.573^∗∗^	0.825^∗∗^
Lack of control				–	0.804^∗∗^
*Cronbach’s α = 0.85*					

*^∗∗^significant correlation (*p* < 0.01).*

**Table 8:** Factor correlation matrix (India).

	Abstinence	Abuse, interference with other activities	Tolerance	Lack of control	TMDbrief
Abstinence	–	0.430^∗∗^	0.642^∗∗^	0.519^∗∗^	0.814^∗∗^
Abuse, interference with other activities		–	0.587^∗∗^	0.532^∗∗^	0.771^∗∗^
Tolerance			–	0.610^∗∗^	0.873^∗∗^
Lack of control				–	0.802^∗∗^
*Cronbach’s α = 0.87*					

*^∗∗^significant correlation (*p* < 0.01).*

In the case of India, only two factors were obtained, each consisting of two factors of the TMDbrief. Specifically, Factor I subsumed Factors I and III of the TMDbrief, and Factor II subsumed Factors II and IV.

### Results Based on Countries

The TMDbrief dependence scores of each country are shown in **Table 9**.

**Table 9:** Mean and SD by countries.

	Sample total			Females			Males		
	Mean	*SD*	*SE*	Mean	*SD*	*n*	Mean	*SD*	*n*
India	21.61	10.97	0.69	21.12	11.11	149	22.33	10.77	100
Ireland	29.71	9.57	0.65	30.28	9.39	113	29.08	9.77	103
Mexico/Guatemala	18.75	9.28	0.53	19.65	9.26	198	17.13	9.15	110
Pakistan	25.23	9.53	0.47	25.32	9.67	192	25.15	9.42	207
Peru	17.20	10.10	0.51	18.84	10.72	198	15.55	9.17	198
Spain	19.07	9.65	0.47	20.40	9.32	284	16.18	9.78	131
Total	21.36	10.61	–	21.91	10.45	1134	20.63	10.78	849

*The English in this document has been checked by at least two professional editors, both native speakers of English. For a certificate, please see: http://www.textcheck.com/certificate/6fUGJJ*

A comparison of the results obtained across countries revealed differences among almost all of them. If we order the dependence scores from highest to lowest, we see that the highest dependence scores were found among Irish young adults, followed in order by those in Pakistan, India, Spain, Mexico/Guatemala, and Peru. All differences were statistically significant, except those between Spain and Mexico/Guatemala (*F*_1,722_ = 0.20, ns); for example: Ireland vs. Pakistan (*F*_1,613_ = 30.87, *p* < 0.001), Pakistan vs. India (*F*_1,646_ = 19.73, *p* < 0.001), India vs. Spain (*F*_1,663_ = 9.69, *p* < 0.01), and Mexico/Guatemala vs. Peru (*F*_1,702_ = 4.40, *p* < 0.05).

**FIGURE 1:**
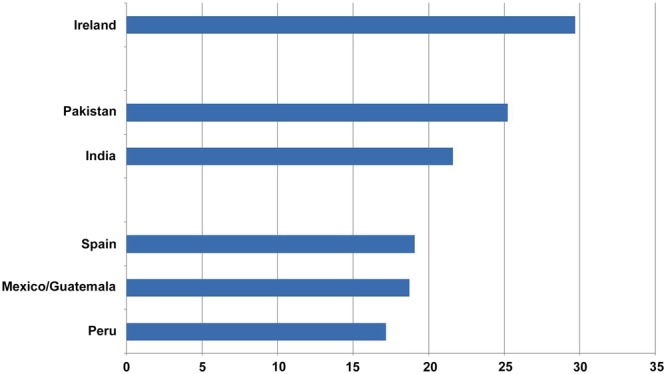
**TMDbrief scores by countries**.

However, the pattern of eta square values was quite different. The values ranged in magnitude from low (eta square from 0.01 to 0.05 for the comparison among Peru, Mexico/Guatemala, Spain, and India and that between Pakistan and India), through medium (eta square from 0.09 to 0.10 for the comparison between Spain and Pakistan and that between Mexico/Guatemala and Pakistan), to high (eta square from 0.13 to 0.27 for the comparisons between Ireland and Peru, Ireland and Mexico/Guatemala, Ireland and Spain, and Ireland and India) (see **Figure 1**).

### Results According to Gender

With regard to the analysis of gender differences, females generally had a higher degree of dependence on mobile phones than did males (**Table 9**). These differences were mainly due to the high scores among females on factors I (*F*_1,1981_ = 10.75; *p* < 0.001) and IV (*F*_1,1981_ = 7.07; *p* < 0.01) (*Abstinence* and *Lack of control*, respectively). No gender differences were observed for *Abuse* or *Tolerance*.

Comparing by country, women had higher dependence scores than did men in Spain (*F*_1,413_ *=* 17.86; *p* < 0.000; η^2^ = 0.04), Mexico/Guatemala (*F*_1,306_ *=* 5.09; *p* < 0.05; η^2^ = 0.02), and Peru (*F*_1,394_ *=* 10.96; *p* < 0.001; η^2^ = 0.03), but there were no significant gender differences in the Pakistani, Indian, or Irish respondents.

## Discussion and Conclusion

Despite obvious differences among communities, societies, and cultures, the use of mobile phones is a global phenomenon. According to data from the International Telecommunication Union, an agency of the World Health Organization that promotes the global development of communication technologies, at present, there are almost as many mobile phones as people in the world (Ict Data and Statistics Division, 2015). In the course of the last 15 years (i.e., from 2000 to 2015), worldwide mobile phone subscriptions have increased from 10 to 97% of the total population. In our sample, four countries (Spain, Guatemala, Ireland, and Peru) had penetration index values >100. India and Pakistan both had penetration index values of approximately 80 (Pakistan Telecommunication Authority [PTA], 2015; Telecom Regulatory Authority of India [TRAI], 2015).

From the outset, many researchers have been concerned with the potential problems that these technologies may cause, especially in younger people (Thomée et al., 2011). One of the most significant problems is addiction, and several instruments have been developed in order to assess this problem (Toda et al., 2006; Chóliz, 2012; Kwon et al., 2013a,b; Lin et al., 2014).

Psychological processes that promote dependence (Griffiths, 2005) are at the core of addiction to technological devices. These processes include tolerance (the escalating need for mobile phone use), withdrawal symptoms (severe emotional disturbance when one cannot use a mobile phone), difficulty controlling use (inability to stop use in many circumstances, even when it is dangerous or inappropriate), familial or social problems due to a dysfunctional pattern of use, and so on. These elements of dependence can be evaluated using questionnaires such as the TMDbrief.

Similar to other behavioral addictions, such as pathological gambling and video-gaming, the inclusion of Internet access has increased the addictive potential of mobile phones because the Internet improves accessibility and availability -two of the main factors contributing addiction (Buljan, 2010)-, with responses to inputs now being almost immediate. The mobile broadband sector is the most important among all broadband sectors, comprising 47% of the market in 2015, representing a 12-fold increase in market share since 2007 (ICT Data and Statistics Division, 2015). This is especially significant because mobile broadband technology allows for the operation of IM systems such as WhatsApp, Snapchat, and others, interpersonal communication systems frequently preferred by young people, as shown in this research. The main issue with these IM systems is that their characteristics can promote dysfunctional use, resulting in the development of dependence.

The main objective of this research was to develop a tool for evaluating the dimensions underlying the addictive process, with respect to mobile phone use among young adults in different countries, to allow for future comparative and cross-cultural studies. In this study, the TMD questionnaire was selected (Chóliz, 2012), and psychometric analyses were carried out, resulting in the development of a short-form questionnaire (TMDbrief) comprising 12 items and representing four factors relevant to the addictive process. Differences in factorial structure between TMD (composed by three factors) and TMDbrief (with four factors) might be due to the fact that the pattern of use of mobiles is quite different between this study and the previous. In the present study, young people use smartphone devices, in which the main applications are the IM systems, whereas before 2011 were mainly phone calls and sms messages. This fact modifies the pattern of use of mobile and, consequently, the associated consequences.

In spite of the differences in factorial structure among TMDbrief and others questionnaires that evaluate the mobile addiction, the main components of addiction which have been extracted in this research are congruent with the other validated tests (Toda et al., 2004; Kwon et al., 2013a,b; Alavi et al., 2014; Demirci et al., 2015). These dimensions, *Abstinence, Abuse, and interference with other activities, Tolerance*, and *Difficulty of control*, are all representative of the addictive process. *Abstinence*, one of the most fundamental characteristics of dependence, refers to the withdrawal symptoms that appear if an addicted person cannot use their mobile phone. This degree of emotional disturbance is not analogous to the normal negative reaction that anyone may experience, but rather is an exaggerated and dysfunctional emotional reaction that does not correspond to the mild inconvenience of temporarily not being able to use a mobile phone. *Abuse and interference with other activities* refers to excessive use of mobiles even in situations where such use is dangerous or inconvenient. The injudicious use of mobile phones can also interfere with other daily activities, such as sleeping or eating; even the individual that is affected may acknowledge that their excessive mobile phone use is harmful. *Tolerance*, the other main dimension of the addictive process, refers, in the present context, to the increasing necessity of mobile phone use. Tolerance is intimately related with excessive use, but it specifically refers to the need for increased use to obtain the same benefits as before. Tolerance also refers to escalating use even in conditions under which one could pursue other activities. The fact that the mobile phone is a multifunctional device biases it toward escalating use, especially with respect to functions related to interpersonal communication. Finally, the *Difficulty of control* dimension was included in the original version of the TMD, as were the other aforementioned dependence criteria. This factor is very common in addictive problems and describes the individual’s inability to stop engaging in the addictive behavior when conditional stimuli associated with that behavio r are presented. Furthermore, addictive behavior often becomes habitual, appearing in numerous different situations.

This factorial structure was replicated for all countries except India. The only thing that varied among these countries was the variance explained by each of the dimensions and, consequently, their extraction order. In the case of India, the four dimensions were grouped into two factors. Nevertheless, these two represented broader dimensions that grouped congruently with the four factors of the TMDbrief: Dimension I subsumed *abstinence* and *tolerance* (Factors I and III of the TMDbrief), and Dimension II subsumed *difficulty of control* and *abuse and interference with other activities* (factors II and IV of TMDbrief). Hence, global scores of the TMDbrief were used to analyze between-sex differences and differences among countries.

Dependence scores obtained for each country differed significantly from one another, with the exception of the scores for Mexico/Guatemala and Spain. If one takes into account effect size, it is possible to group the countries into three separate clusters, such that differences between the countries within each cluster represent small effect sizes, whereas differences between clusters represent moderate or even high effect sizes. These groups are as follows: Group I, consisting of Spanish and Latin American youth; Group II, consisting of Hindu and Pakistani youth; and Group III, consisting of Irish university students.

Differences in levels of mobile dependence were not due to overall patterns of use, because in all cases, participants were university students who used mobile phones on a daily basis, particularly IM systems, and who were familiar with the use of ICT. Moreover, the few researches that compare the pattern of use among different societies show that there are more similarities than differences (Rosenfeld and O’Connor-Petruso, 2014). The observed differences may arise from other cultural factors, representing patterns of cultural affinity among university students within each group, and differences between clusters.

A limitation of this study is that it is not possible to explain the factors responsible for these differences. Our test was sufficiently sensitive to detect differences among several populations with respect to mobile addiction, but to identify and explain these, it will be necessary to design cross-cultural studies in which specific cultural variables are controlled (James, 2014).

Differences among groups were also observed with respect to gender, which is congruent with the evidence that differences occur between male and female when seeking uses and gratifications when using the smartphone (Grellhesl and Punyanunt-Carter, 2012). In general, women had higher scores than did men for mobile phone dependence, but only in the countries belonging to the first cluster (Peru, Guatemala/Mexico, and Spain). No gender differences were found in the Irish, Pakistani, or Indian participants. Such discrepancy among countries may be due to cultural differences that were not considered in this study. Hence, the observed differences should be interpreted with caution, and must be corroborated by well-designed cross-cultural studies. The existing studies are only descriptive in nature and must be validated by future studies controlling for major cross-cultural variables, such as the relevance of gender in social activities, family models and familial relationships, leisure activities, worldview, etc. Nevertheless, the main objective of the present study was not to analyze differences due to country or gender but to develop a brief questionnaire for evaluating mobile phone dependence, with the aim of creating such a tool for use in future cross-cultural studies.

## Author Contributions

MC: main researcher. Design of the research; Sample selection (Spanish population), data collection and global data analysis. Conclusions and Discussion of the results. LP: sample selection (Mexican population), data collection and analysis. SP: sample selection (Indian population), data collection and analysis. EC: sample selection (Irish population), data collection and analysis. AM: sample selection (Pakistani population), data collection and analysis. CF: sample selection (Peruvian population), data collection and analysis. PB: sample selection (Guatemalan population), data collection and analysis.

## Conflict of Interest Statement

The authors declare that the research was conducted in the absence of any commercial or financial relationships that could be construed as a potential conflict of interest.

The reviewer, YW, and handling Editor declared their shared affiliation, and the handling Editor states that the process nevertheless met the standards of a fair and objective review.
